# Central nervous system relapse in a child with anaplastic large cell lymphoma: potential for new therapeutic strategies

**DOI:** 10.1002/cnr2.1377

**Published:** 2021-04-06

**Authors:** Enass H. Raffa, Helen M. Branson, Bo Ngan, Sarah Alexander, Oussama Abla

**Affiliations:** ^1^ Division of Hematology/Oncology, Department of Pediatrics, The Hospital for Sick Children University of Toronto Toronto Ontario Canada; ^2^ Department of Pediatrics, Faculty of Medicine King Abdulaziz University Jeddah Saudi Arabia; ^3^ Division of Neuroradiology, Department of Diagnostic Radiology, The Hospital for Sick Children University of Toronto Toronto Ontario Canada; ^4^ Division of Pathology, Department of Pediatric Laboratory Medicine, The Hospital for Sick Children University of Toronto Toronto Ontario Canada

**Keywords:** ALK, anaplastic, case report, central nervous system, lymphoma, relapse

## Abstract

**Background:**

Central nervous system (CNS) relapse is rare in childhood anaplastic large cell lymphoma (ALCL) and is associated with a poor prognosis.

**Case:**

We describe an 8‐year‐old boy with ALCL who developed an early CNS relapse without initial CNS disease. Despite aggressive medical management, the patient's neurological status deteriorated rapidly and he died shortly after.

**Conclusion:**

Optimal treatment for children with relapsed ALCL involving the CNS remains unclear. Novel agents, including ALK inhibitors, that have CNS‐penetration might be helpful and pediatric studies are warranted.

## 1 INTRODUCTION

Anaplastic large cell lymphoma (ALCL) accounts for 20‐30% of childhood non‐Hodgkin lymphoma (NHL)^1^, ^2^ and often presents with advanced‐stage disease. Disease recurrence develops in 20%‐40% of patients. CNS involvement at the time of diagnosis and relapse remains rare.^3^, ^4^

There is currently no consensus on the optimal treatment for children with ALCL with CNS involvement at presentation and even less clarity on therapy for those with relapsed disease involving the CNS. In this report, we describe a child with ALK+ ALCL with a CNS relapse during first‐line therapy and review the literature on evolving therapeutic options.

## 2 CASE

An 8‐year‐old young boy presented unwell with prolonged fevers, abdominal pain, and progressive respiratory distress. His physical exam was significant for adenopathy and splenomegaly.

Excisional cervical node biopsy revealed a dense proliferation of atypical lymphoid cells and multiple scattered “hallmark cells.” Immunohistochemistry showed diffuse ALK1 expression (nuclear and cytoplasmic), CD30, CD4, focal staining with CD2, and negative CD3 confirming ALK+ ALCL. The pathology met the criteria for the lymphohistiocytic variant.

Diagnostic lumbar puncture (LP) and bone marrow (BM) exam were deferred as the patient could not be sedated due to significant respiratory distress. He was urgently started on therapy according to ALCL99 which includes a 5 day pre‐phase and six alternating courses of intensive chemotherapy derived from the NHL‐BFM protocol.^5^, ^6^ Secondary to his compromised clinical state, intrathecal (IT) chemotherapy due on day 1 of pre‐phase could not be administered. Positive emission tomography (PET/CT), completed 1 week post‐initiation of therapy showed multiple sites of disease including nodal involvement above and below the diaphragm, lung parenchyma, stomach, small bowel, and bilateral kidneys as well as hypermetabolic lytic bone lesions.

Due to his degree of illness including anasarca, high dose methotrexate (HD MTX) was omitted from cycle 1. He clinically improved and was discharged home briefly before the second cycle. LP with triple IT chemotherapy (15 mg MTX and hydrocortisone and 30 mg cytarabine) was given during his second cycle and his cerebrospinal fluid (CSF) analysis was negative at the time.

**FIGURE 1:**
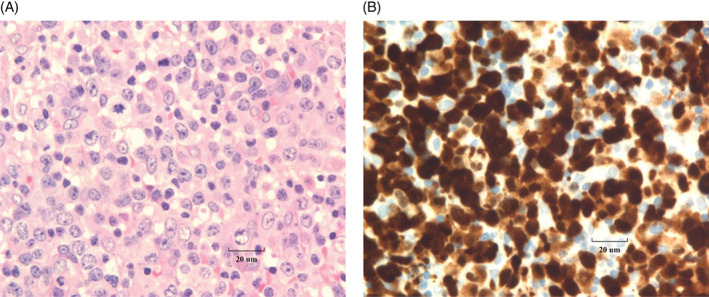
(A) H&E showing the subcapsular and paracortical area of the lymph node replaced by anaplastic large cell lymphoma (ALCL) infiltrate. (B) ALCL cells showed nuclear and cytoplasmic immunostaining for ALK‐1

On the 19th day of his second cycle of chemotherapy, 54 days from diagnosis, he complained of new‐onset headaches, blurry vision, and photophobia. A diagnostics LP showed 135 WBC with blasts. CSF flow cytometry confirmed the presence of ALCL cells that were positive for CD4 and CD30. Magnetic resonance imaging (MRI) showed extensive leptomeningeal enhancement over the cerebral convexities and parenchymal abnormalities within the cerebellar hemispheres consistent with early CNS involvement of ALCL. (Figure 1A,B).

He received triple IT, dexamethasone (10 mg/m^2^/day), and a session of craniospinal irradiation. The following day he was unresponsive and had a generalized tonic‐clonic seizure. Urgent CT showed obstructive tri ventricular hydrocephalus and diffuse extensive leptomeningeal enhancement, particularly in the cerebellum. He received mannitol, hypertonic saline, and an urgent extra ventricular drain (EVD) was inserted. Peripheral blood at that time became positive for circulating blasts, positive for CD30, CD3, and CD4. Ceritinib was started at 20 mg/kg daily through a feeding tube because of its demonstrated CNS efficacy in adult studies.^7^

**FIGURE 2:**
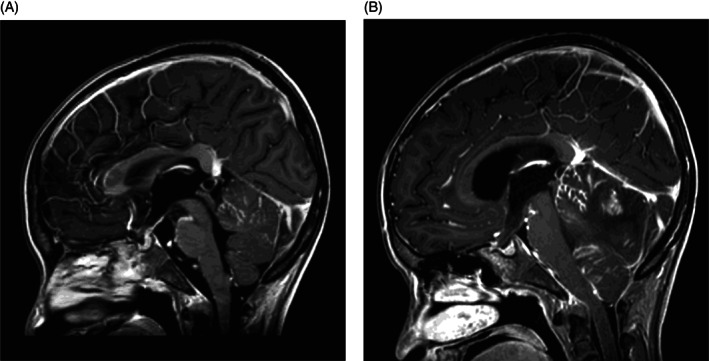
(A) Sagittal post‐Gadolinium T1 image which demonstrates extensive leptomeningeal enhancement in the cerebellar vermis, along the pons and infundibular recess with signal hypointensity in the genu of the corpus callosum. (B) Sagittal post‐Gadolinium T1 image which demonstrates more extensive leptomeningeal enhancement in the cerebellum and vermis which is severely swollen with now effacement of the basal cisterns, compression of the brainstem, and cerebellar tonsillar herniation with hydrocephalus

Subsequent MRI 2 days later showed new cerebellar herniation, compression of the brainstem, and progression of the leptomeningeal enhancement (Figure 2A,B). Despite aggressive medical management, he died 10 days after the date of diagnosis of relapsed disease.

## 3 DISCUSSION

CNS relapses, although rare, contribute to mortality in patients with ALK+ ALCL. Survival chance is 50% with intensive B‐NHL‐type CNS‐directed therapy with or without cranial irradiation.^8^, ^9^

In the patient described, the involvement of the CNS or bone marrow at diagnosis cannot be excluded as these evaluations were not completed. He did have a negative CSF on day 1 of cycle 2 of therapy. He subsequently had clinical, CSF, and radiographic findings of disseminated CNS disease by day 48 of therapy.

Del Baldo et al. described the largest series of patients with CNS relapses in ALK+ ALCL registered on ALCL99 database with an estimated incidence of 4.2% and a 3 year overall survival of 48.7%.^10^ Risk factors specifically associated with CNS relapse include circulating peripheral blasts and bone marrow involvement at diagnosis.^10^

As most clinical trials of de novo ALCL exclude patients with CNS disease, evidence for the efficacy of treatment is lacking both at the time of initial presentation and at relapse. The role of novel agents in the relapsed setting is currently an area of active research. The main limitation in considering these agents for patients with CNS involvement at the time of relapse is whether the agent crosses the blood‐brain barrier (BBB).

Data pertaining to CNS penetrance of these agents are now emerging from a number of clinical trials in adults with ALK+ non‐small cell lung cancer (NSCLC) where 7.5% of patients will have brain metastasis at the time of presentation and 25% to 30% will develop brain metastasis during the course of their illness.^11^, ^12^

Single‐agent vinblastine (VBL) has shown efficacy when given in relapsed ALCL for prolonged durations.^9^, ^13^, ^14^ There has been inadequate evidence of VBL CNS/CSF penetration in high‐risk ALCL relapses. Ruf et al. reported four cases of CNS relapses occurring in patients treated with VBL for a first systemic progression/relapse. They highlight the risk of CNS progression during re‐induction in relapses receiving treatment with limited CNS penetration.^15^

Some patients with CD30+ ALCL treated with Brentuximab vedotin (BV), an anti CD30 chimeric antibody, have subsequently relapsed with CD30‐disease, suggesting a potential mechanism of failure.^16^, ^17^ Its high molecular weight makes it less likely to cross the BBB. Although BV has been shown to induce an objective response in recurrent ALCL,^18^, ^19^ there are reports of CNS progression during re‐induction in relapsed ALCL treated with BV. An adult case report also described isolated CNS relapse despite excellent systemic response to BV.^15^, ^20^

In children, more than 95% of ALCL is ALK+, predominantly due to a translocation NPM‐ALK fusion t(2;5)(p23;q35) making it one of the ideal targets.^21^, ^22^, ^23^ Crizotinib (CRZ), a first‐generation ALK inhibitor, studied in ALK+ pediatric ALCL and in adults with NSCLC showed good responses and prolonged survival.^24^, ^25^, ^26^, ^27^ However, studies have demonstrated low CSF concentrations of CRZ during systemic chemotherapy.^28^, ^29^ CNS relapse or progression while on CRZ treatment have been described,^30^, ^31^, ^32^ even in patients who were initially CNS negative.^33^

Several second‐ and third‐generation TKI (Table 1) have since been developed to overcome the resistance to CRZ as well as manage CNS localizations which has been challenging in adults with NSCLC. Ceritinib, a potent oral second‐generation ALK inhibitor, has been shown to have 20‐fold greater potency than CRZ and highly effective against common CRZ associated mutations.^34^ In phase II ASCEND 2 trial, ceritinib achieved intracranial responses in patients with baseline brain metastasis. Subsequent data from ASCEND 8 showed similar efficacy and tolerable gastrointestinal toxicity with reduced dosing.^35^, ^36^, ^37^ Data is still lacking in the pediatric population. Final analysis from a pediatric phase I study (NCT01742286) in pediatric patients with advanced, mostly pre‐treated, ALK‐aberrant malignancies showed the toxicity profile is similar to that in adults. Of note, out of the 55 patients treated with ceritinib, eight had a diagnosis of ALCL. The overall response rate (ORR) (95% CI) was 75% for the patients with ALCL.^38^

**TABLE 1:** CNS efficacy and toxicity reported with different generation ALK inhibitors

ALK inhibitors	Target kinase	CNS penetrance	Clinical Trial	CNS efficacy in adults with ALK+ NSCLC	Toxicity	Pediatric trials/cases	References
Crizotinib	ALK c‐MET ROS1	Poor	PROFILE 1005 and 1007 PROFILE 1014 ALEX NCT02075840	CNS progression on crizotinib in 72% of patients with ALK+ NSCLC Intracranial time to tumor progression not significantly different between crizotinib vs chemotherapy arm CNS progression in 45% of patients in treatment naiive ALK+NSCLC	Neutropenia, lymphopenia, elevated ALT, and hypophosphatemia	Phase I/II trial NCT00939770: CNS metastasis/tumors excluded after two patients had intratumoral hemorrhage (Mosse 2013) Ruf et al. (2018): Case series: 2 patients with CNS progression on crizotinib Mosse et al 2017:26 patients with R/R ALCL. CNS status not given	15, 25, 26, 28, 29, 61, 62, 63, 64
Ceritinib	ALK IGR‐1R INSR STK22D	Yes	ASCEND‐1 to 5 ASCEND‐ 4 ASCEND‐7	Reported intracranial responses in pts with measurable baseline brain lesions in ALK+ NSCLC Overall IC‐RR was 57% with ceritinib vs 22% with chemotherapy Median PFS: 5.2 months and the median OS: 7.2 months.	Vision disorder, bradycardia, interstitial lung disease/pneumonitis, hepatotoxicity, and renal failure	Phase I study NCT01742286: no CNS data	7, 35, 65
Alectinib	ALK LTK GAK	Yes	ALEX trial	CNS progression under crizotinib in 45% of cases vs 12% with alectinib, OS benefit in pts with CNS metastasis	Elevated ALT, elevated AST, elevated creatinine, anemia, pneumonia	Phase II UMIN000016991: 10 pts with R/R ALCL. 1 year PFS 58.3%, EFS 70% and OS 70%. Pts with CNS disease excluded	47, 63, 66
Brigatinib	ALK ROS1	Yes	ALTA 1 L	CNS progression 9% brigatinib vs 19% with crizotinib IC‐ORR observed in 53% of pts, median IC‐PFS was 14.6 months.	Fatigue, diarrhea, visual disturbance, pneumonia, interstitial lung disease/pneumonitis	None	49, 67
Lorlatinib	ALK ROS1	Yes (CSF drug concentrations 75% of plasma levels)	Phase II NCT01970865	IC‐ORR 66.7% in treatment‐naive patients and 63% in pre‐treated	Hypercholesterolemia, hypertriglyceridemia, edema, peripheral neuropathy and central nervous system effects	NANT 2015‐02 NCT03107988: Recruiting	37, 51

Abbreviations: ALT, alanine aminotransferase; AST, aspartate transaminase; IC‐ORR, intracranial objective response rate; IC‐PFS, intracranial progression‐free survival; OS, overall survival; PFS, progressionfree survival; R/R, relapsed, refractory.

Alectinib, another second‐generation highly selective inhibitor, has been shown to have activity against L1196M, a common mutation causing CRZ resistance.^39^ Based on the ALEX study of ALK+ NSCLC with brain metastasis, alectinib showed a higher ALK inhibitory potency,^40^ BBB transport, superior CNS activity, and significantly delayed CNS progression, irrespective of prior CNS disease or radiotherapy when compared to CRZ.^41^, ^42^, ^43^, ^44^ The excellent intracranial control translated into survival benefit.^45^ Results from a phase II trial evaluating alectinib in refractory/relapsed ALCL in 10 patients, four of which were pediatric patients, showed favorable clinical activity where eight out of 10 patients achieved complete response.^46^, ^47^

Brigatinib, another second generation, dual inhibitor of ALK and EGFR, showed activity in NSCLC with CNS lesions in an early phase I/II trials.^48^ In the phase III ALTA‐1L trial, brigatinib also demonstrated superior intracranial efficacy as compared to CRZ.^49^

Lorlatinib is a third‐generation ALK inhibitor designed to have a pan‐inhibitory activity against ALK. In phase I, NSCLC study response rates with lorlatinib in patients with measurable and non‐measurable brain metastases reached 39% and 31%, respectively.^50^ In the phase II study, lorlatinib yielded intracranial overall response rates of 66.7% in treatment‐naive patients with measurable brain metastases and 63% in those treated with at least one ALK inhibitor.^51^

Other alternatives include the use of immune checkpoint inhibitors. Programmed death ligand 1 (PD‐L1), whose expression is induced by NPM‐ALK to promote immune evasion by STAT3 pathway activation, is being targeted. Nivolumab, a PD1 inhibitor, showed a prolonged response in patients with refractory ALK+ ALCL.^52^, ^53^ It was also reported to have a good response activity in adults with primary CNS lymphoma.^54^

CD30 is a promising target universally expressed in all ALCL among other lymphomas. Two recent clinical trials of CD30‐directed Chimeric Antigen Receptor T (CAR‐T) cells in relapsed/refractory (r/r) CD30+ lymphomas have shown preliminary efficacy in patients with heavily treated r/r disease.^55^, ^56^, ^57^, ^58^ Frigault et al. described eight patients with secondary CNS lymphoma treated with tisagenlecleucel where the activity of CAR T cells within the CNS space was demonstrated.^59^ Barriers still to overcome for CNS efficacy include an immune‐suppressive microenvironment, unique properties to the CNS that limit T cell entry, and risks of immune‐based toxicities in this highly sensitive organ.^60^

In summary, we describe a child with an early CNS relapse of ALK+ ALCL who died despite aggressive management. Optimizing CNS‐directed therapy for children with ALCL both in initial therapy and at the time of relapse deserves further research. Multiple agents in development may have an important role in this setting.

## AUTHOR CONTRIBUTIONS

**Helen Branson:** Resources. **Bo‐Yee Ngan:** Resources. **Sarah Alexander:** Supervision; writing‐review & editing. **Oussama Abla:** Supervision; writing‐review & editing.

## CONFLICT OF INTEREST

No potential sources of conflict of interest.

## ETHICAL STATEMENT

Institutional approval was not required for a case report. All the patient information was de‐identified for the purpose of this case report. Patient consent was therefore not obtained for publication.

## Data Availability

The data that support the findings of this study are available on request from the corresponding author. The data are not publicly available due to privacy or ethical restrictions.
